# Identification of Important Chemical Features of 11β-Hydroxysteroid Dehydrogenase Type1 Inhibitors: Application of Ligand Based Virtual Screening and Density Functional Theory

**DOI:** 10.3390/ijms13045138

**Published:** 2012-04-23

**Authors:** Sugunadevi Sakkiah, Chandrasekaran Meganathan, Young-Sik Sohn, Sundaraganesan Namadevan, Keun Woo Lee

**Affiliations:** 1 Division of Applied Life Science (BK21 Program), Systems and Synthetic Agrobiotech Center (SSAC), Plant Molecular Biology and Biotechnology Research Center (PMBBRC), Research Institute of Natural Science (RINS), Gyeongsang National University (GNU), 501 Jinju-daero, Gazha-dong, Jinju, 660-701, Korea; E-Mails: suguna@bio.gnu.ac.kr (S.S.); megac@bio.gnu.ac.kr (C.M.); ysohn@bio.gnu.ac.kr (Y.-S.S.); 2 Department of Physics (FEAT), Annamalai University, Annamalainagar 608 002, Tamil Nadu, India; E-Mail: sundaraganesan_n2003@yahoo.co.in

**Keywords:** 11β-hydroxysteroid dehydrogenase, pharmacophore, density function theory, molecular docking, diabetes, virtual screening

## Abstract

11β-Hydroxysteroid dehydrogenase type1 (11βHSD1) regulates the conversion from inactive cortisone to active cortisol. Increased cortisol results in diabetes, hence quelling the activity of 11βHSD1 has been thought of as an effective approach for the treatment of diabetes. Quantitative hypotheses were developed and validated to identify the critical chemical features with reliable geometric constraints that contribute to the inhibition of 11βHSD1 function. The best hypothesis, Hypo1, which contains one-HBA; one-Hy-Ali, and two-RA features, was validated using Fischer’s randomization method, a test and a decoy set. The well validated, Hypo1, was used as 3D query to perform a virtual screening of three different chemical databases. Compounds selected by Hypo1 in the virtual screening were filtered by applying Lipinski’s rule of five, ADMET, and molecular docking. Finally, five hit compounds were selected as virtual novel hit molecules for 11βHSD1 based on their electronic properties calculated by Density functional theory.

## 1. Introduction

Glucocorticoids are a class of steroid hormones which bind to the glucocorticoid receptor. The glucocorticoids name was derived from their role in the regulation of glucose metabolism and from their synthesis. Glucocorticoids play an important role in the regulation of multiple physiological processes such as energy metabolism, maintenance of blood pressure, stress responses, and cognitive functions. The dysregulation of glucocorticoids has been implicated in the pathogenesis of diabetes and metabolic syndromes. The major glucocorticoid, cortisol, is normal in healthy individuals but the increased level in the intracellular adipose tissue could be responsible for “visceral obesity”. Cortisol is an important regulator of fuel metabolism during the starvation and stress which is modulated by 11β-hydroxysteroid dehydrogenase type 1 (11βHSD1) and 11β-hydroxysteroid dehydrogenase type 2 (11βHSD2). 11βHSD1, a NADP(H)^+^-dependent bidirectional enzyme, was highly expressed in the liver [1], adipose tissue [2] as well as found in a number of other tissues such as brain [3], blood vessels [4], macrophage [5], eye [6], bone [7], lung [8], and ovary [9]. 11βHSD1 regulates the access of glucocorticoid to steroid receptors by catalyzing the conversion of inactive cortisone into active cortisol. 11βHSD2 is a NAD^+^-dependent dehydrogenase which catalyzes the reverse reaction of 11βHSD1 (converts the active cortisol into inactive cortisone). 11βHSD2 is mainly localized in the mineralocorticoid receptor (MR), and target tissues like kidney, colon, and salivary gland where it functions to protect the MR from excessive exposure to cortisol.

**Figure 1:**
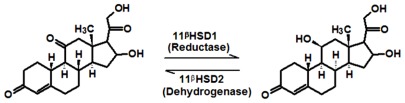
Enzymes involved in the conversion of inactive cortisone to active cortisol.

11βHSD1 and 11βHSD2 share a high similarity with other members of the short chain dehydrogenase/reductase (SDR) family. Both the enzymes are functionally quite different and catalyze the interconversion of glucocorticoids (Figure 1). The SDR family enzymes shares a conserved α/β nucleotide-binding Rossman fold which consists of five parallel β-sheets flanked by three α-helices on the right and left side each. SDR reactions are catalyzed by a Tyr-(Xaa)3-Lys motif, often combined with a conserved Ser which helps to orientate the substrates in a suitable position. From all the reported 11βHSD1 crystal structures we observed that the co-crystal ligand showed a close vicinity to the co-factor as well as the catalytic amino acid Tyr183. The ligand orientation in the active site of 11βHSD1 was well stabilized by the strong interaction with Ser170.

Elevated levels of 11βHSD1 develop the metabolic syndrome-like phenotypes and also resist weight gain on a high-fat diet which is associated with increased energy expenditure and improved glucose tolerance as well as insulin sensitivity [10]. There are several factors which regulate the 11βHSD1 function such as hormones, transcriptional factors, co-factors, growth factor, tumor necrosis factor-β, and gonadal steroids [11]. Hence, 11βHSD1 inhibition could be a promising approach for the treatment of diabetes, cardiovascular, and metabolic syndromes [12–14]. The metabolic activation of cortisol is by one of the glucocorticoid hormones from a cortisone precursor and is exclusively carried out by 11βHSD1. The specific inhibition of 11βHSD1 emerges as a promising novel drug target in metabolic disease and other glucocorticoid-related syndromes due to the central role of cortisol in metabolic syndromes like obesity, insulin resistance, dyslipidemia, and arterial hypertension. Unregulated overexpression of cortisol, manifested in Cushing’s disease is genetic evidence for the role of 11βHSD1 in diabetes and obesity. Suppression of 11βHSD1 function may retard the development of antherosclerosis and hence it acts as a potential treatment for metabolic syndrome, diabetes, and cardiovascular disease. Therefore, inhibitors of 11βHSD1 are very attractive to the pharmaceutical industry and researchers.

In recent years, several classes of 11βHSD1 inhibitors have been disclosed. In 2002, Barf *et al*. [15] reported 2-aminothiazole sulfonamides as the first selective 11βHSD1 inhibitors. Yang *et al.* [16] described the important chemical features from a structure-based hypothesis, as well as highlighting that the hydrogen bond interaction between the ligand and Tyr183 or Ser170 plays a crucial role in the 11βHSD1 inhibition. Ligand-based pharmacophore modeling is one of the productive tools to identify the important chemical features of the inhibitor as well as to improve its potency and pharmacokinetic properties. In this work, the known 11βHSD1 inhibitors were collected from the literatures to generate and validate the 3D pharmacophore models. The reported structure-based pharmacophore models have been compared with our ligand-based pharmacophore model to select the important chemical features responsible for inhibiting the 11βHSD1 function. A hypothesis was developed based on the reported inhibitors of 11βHSD1 and the best hypothesis was used to screen several databases as an initial filtration in virtual screening. The screened molecules were subjected to a molecular docking study to find the suitable orientation and hydrogen bond interactions between the lead compounds and the active residues such as Try183 and Ser170. Orbital energy values were calculated to find the reactivity of the lead compounds by applying density functional theory (DFT).

## 2. Results and Discussion

Pharmacophore modeling is a widely utilized method in the computer-aided drug design process. Within this framework two major domains are covered: virtual screening for a new lead which is nothing but a “scaffold hopping”; and systematization of activity distribution within the group of molecules, displaying a similar pharmacological profile that is recognized by the same target. The 3D pharmacophore modeling was used to identify the critical chemical features of 11βHSD1 inhibitors. The best hypothesis model was selected and validated based on its predictability in terms of activity and used to guide the rational design of 11βHSD1 inhibitors.

### 2.1. Pharmacophore Generation

**Figure 2:**
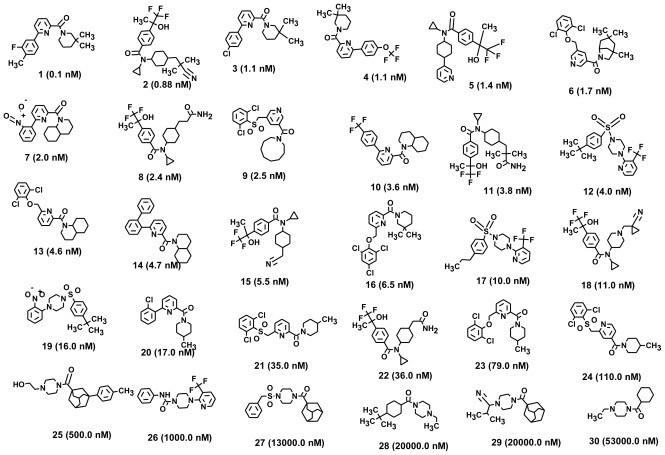
Thirty chemically diverse compounds with their IC_50_ values in brackets used as training set in 3D-QSAR Discovery Studio/Pharmacophore generation.

The selection of chemical features plays an important role in determining the hypothesis quality. Yang *et al.* in 2008 reported a quantitative hypothesis of six features which consists of L-hydrogen bond acceptor (HBA), 1-ring aromatic (RA), and 4-hydrophobic (Hy) chemical features. Hence, these chemical features were selected based on the reported quantitative ligand-based pharmacophore models. During the development of pharmacophore models generation, the training set molecules (Figure 2) were mapped to the chemical features in the hypothesis with their predetermined conformations which were generated using the Best conformation module. The pharmacophore generated ten alternative hypotheses based on the reported IC_50_ values of 11βHSD1 inhibitors. All hypotheses include chemical features such as HBA, RA, and hydrophobic aliphatic (Hy-Ali), hence these chemical features were assumed to be critical for the inhibition of 11βHSD1 function. Among ten hypotheses, one hypothesis was chosen as a best pharmacophore model based on its statistical parameters such as highest correlation coefficient, good cost difference, and lowest RMSD.

#### 2.1.1. Selection of the Best Hypothesis by Debnath Analysis

The quality of the generated pharmacophore model is best described in terms of fixed cost, null cost, and total cost defined by Debnath [17]. The fixed cost stands for an ideal hypothesis that perfectly fits the estimated and experimental activity values with minimum deviation. The null cost represents the cost of a hypothesis with no features that estimates activity to be average [18]. The difference between the fixed and null cost should be greater or equal to 60 bits. The highest value indicates a greater chance of finding a useful hypothesis and also reflects the chance correlation. In this study, the cost difference for all ten hypotheses was higher than 60 bits which represented the 90% statistical significance of the pharmacophore models. Hypo1 was believed to be statistically relevant and selected as a best hypothesis based on the following criteria, such as the highest cost difference (157.30), lowest error cost (117.67), the lowest RMS (1.21) divergence, and the best correlation coefficient (*r*:0.94) (Table 1). Perceptibly, all the above results demonstrated that Hypo1 was a reliable hypothesis with a good predictive power.

#### 2.1.2. Score Hypothesis

**Figure 3:**
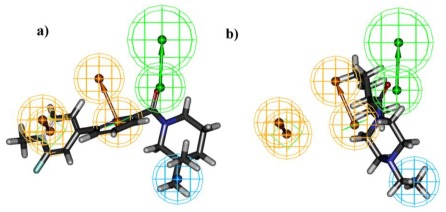
Best pharmacophore model Hypo1 aligned to training set compound (**a**) Active molecule compound 1 (IC_50_ 0.1 nM); (**b**) Inactive molecule compound 18 (IC_50_ 53000 nM). Pharmacophore features are color coded (Hy-Ali, hydrophobic aliphatic, blue; RA, ring aromatic, brown and HBA, hydrogen bond acceptor, green).

**Figure 4:**
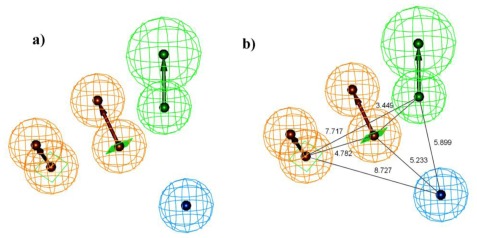
HypoGen pharmacophore model Hypo1 and its geometric constrains, where RA (ring aromatic), Hy-Ali (hydrophobic aliphatic) and HBA (hydrogen bond acceptor) are illustrated in brown, blue and green, respectively.

All the training set compounds were classified into three categories based on their IC_50_ values such as highly active, moderately active, and low active. Hypo1 was used to screen the training set to anticipate as well as to evaluate the predicted and experimental activity value of the compounds (Table 2). All the training set compounds showed an error value of less than ten which indicates only one order of magnitude of difference was observed in the discrepancy between the predicted and experimental activity. This variation may be due to the difference in the number of degrees of rotation of molecules which mismatched with Hypo1. Considering the above evaluations, Hypo1 was selected as a reliable best pharmacophore model to describe the structure activity relationship of 11βHSD1 inhibitors. The most active compound 1 (IC_50_: 0.1 nM) showed a fitness score value of 9.37 when mapped to Hypo1, whereas the least active compound 30 (IC_50_: 53,000 nM) showed a fit value of 5.38 (Figure 3). This result indicates that Hypo1 consists of a reasonable pharmacophoric characteristic of 11βHSD1 inhibitors. Hence the small molecules which satisfy the chemical features of Hypo1 such as one HBA, one Hy-Ali, and two RA chemical features with the specific geometric orientation (Figure 4) will be good inhibitors for 11βHSD1.

### 2.2. Pharmacophore Validation

#### 2.2.1. Fischer’s Randomization Test

**Figure 5:**
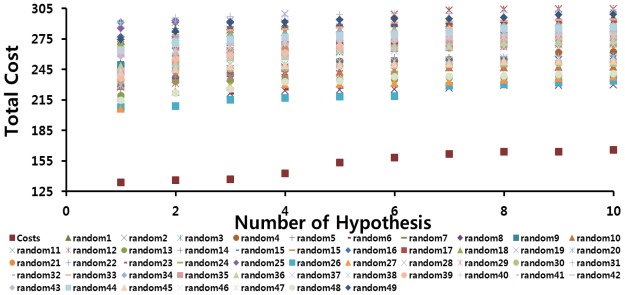
The difference in costs between HypoGen runs and the scrambled runs. The 98% confidence level was selected.

Fischer’s test was applied to evaluate the statistical significance of Hypo1 based on statistical validation. In this method the experimental activity values of training set compounds were scrambled randomly and subsequently the scrambled datasets were used to generate the number of hypotheses depending on their significant levels. All the parameters were set as in the initial hypothesis generation. This validation was to check if there was any strong correlation between the structures and activity values. For this study, we selected the 98% confidence level; hence 49 random spreadsheets were produced by mixing up the experimental activity values present in the training set. By analyzing the resultant 49 hypotheses, Hypo1 was shown to be far superior to the 49 random pharmacophore models (Figure 5). This cross validation confirmed that there was a 98% chance of the pharmacophore model representing a true correlation in the training set activity data and thus gave us strong confidence in Hypo1.

#### 2.2.2. Test Set Method

A good pharmacophore model should predict the correct activity range of independent compounds present in the test set. The test set, containing 20 structurally different molecules from the training set, was used to validate the predictability of the Hypo1. All the test set compounds were prepared in the same way and classified into three categories as training set compounds such as highly active, moderately active and low active molecules. In general, a compound with the highest fit value corresponds to a compound which has a good activity value. In analyzing the error value of the test set, we found that all error values were less than two and the regression analysis of experimental and predicted inhibitory activity values (Table 3) gave a fairly good correlation coefficient of 0.93 which indicates good predictive ability of Hypo1. Therefore, it is obvious that Hypo1 is capable of quantitatively distinguishing between highly, moderately, and low active compounds.

#### 2.2.3. Decoy Set

Finally, a decoy set was generated to evaluate the efficiency of Hypo1 using a Ligand Pharmacophore Mapping module by computing GH and EF. The decoy set contains 13 active 11βHSD1 inhibitors and 1287 inactive or the unknown activity compounds for 11βHSD1. The total number of compounds in the database (D) 1300, 12 compounds in the hit list (H_t_), 75% active yields (%Y), 69.23% ratio of actives in the hit lists (%A), values of EF (7.5) and GH (0.73) are all a very good indication of the high efficiency of Hypo1 (Table 4). From the overall validations, we were assured that Hypo1 can predict most of the estimated and experimental activity of molecules in the same order of magnitude as well as it can discriminate the active inhibitors from inactive compounds as confirmed by the test and statistical parameters of the decoy sets, respectively.

Initially the best quantitative hypothesis, Hypo1, was compared with the reported structure based hypothesis of Yang *et al*. The refined structure-based pharmacophore model consists of 1-HBA, 1HBD, and 4-Hy chemical features and our best hypothesis (Hypo1) consists of 1-HBA, 2-RA, and 1-Hy chemical features. The difference between these two hypotheses may be due to molecular structure diversity. Hypo1 lacks the HBD chemical feature which was mentioned, in that it forms strong interactions between the ligand and its cofactor. The same group in 2008 developed a quantitative hypothesis which consisted of 4-Hy, 1-HBA, and 1-RA groups. Interestingly, all these three different types of hypotheses indicated that the HBA, Hy chemical features play a critical role in the inhibition of 11βHSD1.

### 2.3. Pharmacophore Model Based Virtual Screening

**Figure 6:**
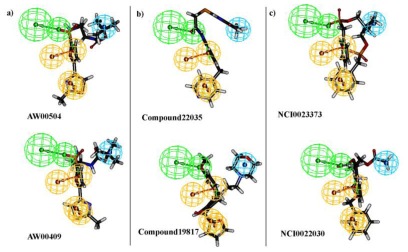
Mapping of hit molecules by Hypo1 from chemical databases. (**a**) Maybridge; **(b**) Chembridge; (**c**) NCI. Pharmacophore features are color coded Hy-Ali, hydrophobic aliphatic, blue; RA, ring aromatic, brown and HBA, hydrogen bond acceptor, green.

The best hypothesis, Hypo1, was used as a 3D query for retrieving potent compounds from the chemical databases such as NCI, Maybridge, and Chembridge. As a first screening, Hypo1 retrieved 6,378, 20,021, and 13,470 compounds from NCI, Maybridge, and Chembridge, respectively. To sort out these molecules, a maximum fit value of 11 was applied which reduced the number of molecules to 375 from NCI, 1192 from Maybridge and 440 from Chembridge. These hit molecules were further sorted by applying the Lipinski’s rule of five and ADMET to give them more drug-like properties. According to the rule of five, compounds are considered likely to be well absorbed when they possess LogP of less than five, molecular weight less than 500, number of HBD less than five, number of HBA less than ten, and number of rotatable bonds less than ten. The molecular flexibility of molecules and the total number of HBAs and HBDs are found to be important predictors for a compound to have a good oral bioavailability. The ADMET functionality was used to estimate the values of Brain Blood Barrier (BBB) penetration, solubility, Cytochrome P450 (CYP450) 2D6 inhibition, Hepatotoxicity, Human intestinal absorption (HIA), Plasma Protein Binding (PPB), and to access a broad range of toxicity measure of the ligands. Among all these criteria, we mainly focused on BBB (cut-off value 3), solubility (cut-off value 3), and HIA (cut-off value 0) because these are important criteria for a compound to have a good oral bioavailability drug. Totally, 165 compounds (73 NCI, 65 Maybridge, and 27 Chembridge) have satisfied all the physiological properties to be ideal lead molecules (Figure 6). Thus, these molecules were subsequently subjected into the molecular docking to reduce the false positive rate.

### 2.4. Molecular Docking

**Figure 7:**
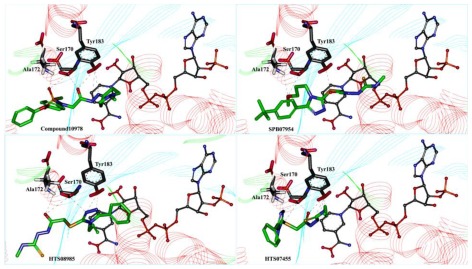
The orientation of hit molecules in the active site of 11βHSD1. Green color—leads and hydrogen bonds in black are shown.

**Figure 8:**
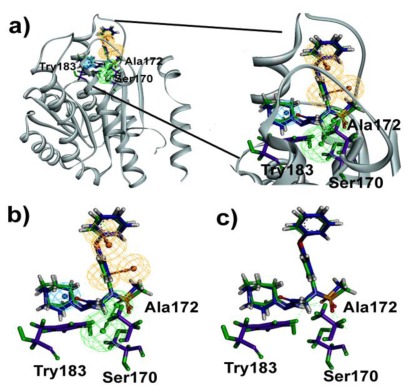
(**a**) Compound 10978 fit with Hypo1 was overlaid in the active site of 11βHSD1 crystal structure (PDB ID: 3REI) represented as ribbon; (**b**) overlay of compound 10978 with docked pose and geometric constraint of Hypo1; (**c**) overlay of compound 10978 with docked pose and geometric constraint of Hypo1 without the hypothesis. Pharmacophore features are color coded (Hy-Ali, hydrophobic aliphatic, blue; RA, ring aromatic, brown and HBA, hydrogen bond acceptor, green). Green color molecules represent the Hypo1 fit molecule and violet color represents the dock pose.

Biomolecular interactions and binding properties were analyzed for the hit molecules from the virtual screening using GOLD molecular docking software. Three dimensional complex structure of 11βHSD1 (PDB ID: 3FRJ) was taken from the PDB as a receptor. Initially the co-crystal was docked in the active site of 11βHSD1 to check whether the selected parameters are able to produce the most suitable binding orientation. The result revealed that GOLD can perfectly reproduces the similar orientation of the co-crystal. Then, GOLD was used to refine the retrieved 165 hit molecules by docking into the inhibitor binding site of the 11βHSD1 and the Gold fitness score was used to select the docked compounds. The well docked small molecules with lowest docked energy as well as average Gold fitness score were enumerated. The active molecules from the training set shows a Gold fitness value in the range of 61–70 but the moderately active molecules shows a value lesser than 60. Thus a Gold fitness score value of greater than 60 was considered as the cut off value to select the docked hit compounds from the virtual screening. The most stable docking models (47 compounds) were selected according to the best-scored conformation by the Gold fitness score function. The molecules which show the greater value were manually checked for the hydrogen bond interactions with the critical amino acids like Tyr183, Ser170, and Ala172 of 11βHSD1. Among these molecules, 17 molecules had shown good hydrogen bond interactions with Tyr183, Ser170 or Ala172 (Figure 7). The remaining 30 molecules had shown good interactions with the active residues Tyr183 but failed to show hydrogen bond interactions with Ser183 or Ala172. The compound 10978 was overlaid to check its docked pose orientation and the geometric constraints of Hypo1 (Figure 8). Finally, 17 compounds (Table 5) have satisfied all the above filtering methods and all the critical chemical features of Hypo1, docking score, and drug-like properties.

### 2.5. Density Functional Theory

The DFT has been applied to find the orbital energy values from which we can predict how well the group can donate or accept the electrons. Recently, many research articles reported the donating and accepting ability of the small molecules by calculating the highest occupied molecular orbital (HOMO) and lowest unoccupied molecular orbital (LUMO) values [19]. The most active 3 training set molecule classified based on their IC_50_ value such as Compound 9 (2.5 nM), Compound 12 (4 nM), Compound 17 (10 nM) along with 17 database hit compounds were taken into account of HOMO, LUMO calculation. The calculated HOMO, LUMO values of hit compounds were compared with the most active training set compounds to analyze their electronic properties such as electron donating and accepting toward 11βHSD1. The frontier orbital, HOMO, is an important parameter of molecular electron structure. The higher HOMO value implies that the molecule has good electron donating ability; on the other hand, a lower value implies weak electron donating ability. The calculated HOMO, LUMO values have been summarized in Table 6.

**Figure 9:**
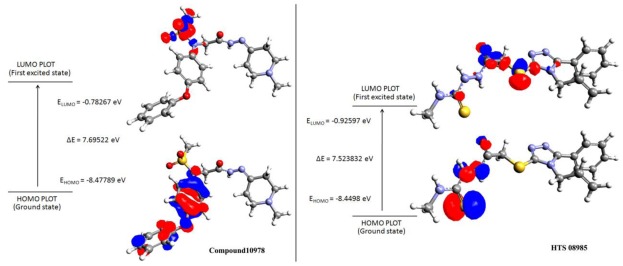
Highest and lowest molecular orbital plots for two lead compounds identified from the databases.

In comparison with training set compounds 4, compounds from Maybridge (KM06091, HTS08985, HTS07455, SPB07954), and one compound from Chembridge (Compound 10978) have shown very high HOMO values. This demonstrates that the hit compounds have more electron donating ability than most active training set compounds. Moreover, HOMO-LUMO energy gap of training set and hit compounds were calculated to check the chemical activity of the molecules suitable to donate or accept electrons towards 11βHSD1. Further, the lowering of the energy gap demonstrates the eventual charge transfer interaction that takes place within the molecule [19]. In the present study, the five hit compounds showed lower energy gap values when compared with the most active training set compounds. The atomic orbital compositions of the frontier molecular orbital for some of the hit compounds such as HTS08985 and Compound 10978 were sketched and shown in Figure 9. In the case of HTS08985, the highest molecular orbitals were located mainly for thiomethane and lowest for methylsulfonyltriazole. Hence the electronic transition from thiomethane to methylsulfonyltriazole and this result was confirmed by the molecular interaction of the compound in the protein active site (Figure 7). Compound 10978 has also shown similar results which was also confirmed by the molecular interaction. Based on the above results, the database hit compounds are more superior to most active compounds and could be used to design new classes of 11βHSD1 inhibitors.

## 3. Experimental Section

### 3.1. Pharmacophore Modeling

Pharmacophore modeling is one of the most potent and rapid method to discover the novel scaffolds from databases. Pharmacophore can be produced by two methods: based on the active site of protein and also the properties of the ligands. The ligand based pharmacophore exists in two different approaches: (i) based on common features of the molecules; and (ii) using the activity values and the structure of the compounds. In this study we employed the activity based pharmacophore model generation method.

Software: A pharmacophore was generated using HypoGen module in Accelrys Discovery Studio v2.5 (DS, Accelrys, San Diego, CA, USA, 2011). The two- and three-dimensional structures of compounds were constructed using Chemsketch v11.02 and DS, respectively.

#### 3.1.1. Training and Test Set Preparation

To construct training and test sets, totally we selected 50 structurally diverse compounds with the reported inhibitory activity values from literatures [20–26]. The constructed pharmacophore model can be as good as the information of input data. Hence, to achieve a best hypothesis, the training set must-obey the rules in a 3D quantitative structure-activity relationship generation. The training set must consist of widely populated (at least 16 compounds) as well as it should have good structural diverse representatives covering an activity range of at least 4 orders of magnitude. The most active compounds should inevitably be included in the training set and all biologically relevant data should be obtained by homogeneous procedures and expressed as IC_50_ (*i.e*., concentration of compound required to inhibit 50% of 11βHSD1). All the structures were minimized using smart minimizer algorithm which performs 1000 steps of steepest decent with a root mean square [1] gradient of 0.1, followed by conjugated gradient minimization. Among 50 molecules, 30 compounds were selected as training set and the remaining 20 compounds were taken as test set. The training set was selected based on the structural diversity and wide coverage of the activity values which spans across a wide range from 0.10 nM to 53,000 nM. In this modeling study, based on the activity values (IC_50_) the compounds are divided into three categories: compounds which have the activity values of <10 nM were classified as highly active (+++), those with activities between ≥10 nM and ≤100 nM were defined as moderately active and the compounds that have the activity values greater than 100 nM was classified as low active molecules. The significant aspect of this assortment to ensure that each active provide a clue to generate the hypothesis thus it can be able to bring out as much as vital information possible for predicting biological activity.

#### 3.1.2. Pharmacophore Generation

DS provides two types of conformational analyses such as Fast and Best. Molecules might adjust their conformations when binding to a receptor hence the conformers was generated for each molecule to increase the flexibility during the generation of the pharmacophore. Conformational models of the training and test set compounds were generated using a Monte Carlo-like algorithm together with poling [27,28]. Instead of using lowest energy conformation of each compound, multiple acceptable conformations were generated for each compound specifying the maximum number of 255 conformers with a constraint of 20 kcal/mol energy and all other default parameters were used.

For the pharmacophore studies, the following chemical features were selected using Feature mapping, to get the essential information for hypothesis generation process: HBA, HBD, RA, Hy-Ali, positive ionization (PI), negative ionization (NI), and hydrophobic aromatic (Hy-Ar). The uncertainty factor for each compound represents the ratio of uncertainty in activity value based on the expected statistical straggling of biological data collection. The 30 training set molecules which are associated with their conformations are submitted to 3D QSAR Pharmacophore Generation. It produces the hypothesis by undergoes three phases: constructive, subtractive, and optimization phases. The initial stage of the hypothesis generation is constructive phase which considers all possible pharmacophore configurations of the most active compounds to entail pharmacophore demands. Second phase is a subtractive phase, all possible pharmacophore configurations of the remain or discarded depending on the number of least active training set members that share a pharmacophore pattern. Finally, the optimization phase, candidate model within the pharmacophore space of a particular target through fine perturbation to pharmacophore hypotheses is evaluated. The hypothesis generation process stops when no better score of the hypothesis can be accomplished and the output parameters also determine the hypothesis quality. The regression parameter in pharmacophore generation module estimates the activity of each training set which computed by the regression analysis using the relationship of geometric fit value versus the negative logarithm of activity. The geometric fit value was calculated based on the fit function checking whether the feature is mapped or not and also assures whether it contains a distance term, which measures the distance that separates the feature on the molecule from the centroid of the hypothesis feature. Finally, the top 10 scoring hypotheses were exported which composed of above mentioned pharmacophore features. The best model was selected based on the statistical parameters like cost values which determine the significance of the model. The best model was further affirmed by various validations methods like Fischer’s randomization method, test and decoy sets.

### 3.2. Methods to Select the Best Pharmacophore Model and to Validate the Hypothesis

Among the 10 hypothesis, the selected best hypothesis should be able to identify the active compounds from external molecules (other than training set) and also to predict the activity of the molecules accurately. Therefore Debnath’s analysis was used to select the best hypothesis, Fisher’s randomization test method, test and decoy sets were used to confirm the predictive ability of the best hypothesis.

#### 3.2.1. Debnath’s Analysis

The success of any hypothesis is determine by two important theoretical cost calculations like fixed and null cost. Fixed cost also known as ideal cost that determines the simplest model which can perfectly fits all the data. Null cost also termed as no correlation cost, representing the highest cost of a pharmacophore with no features and estimates activity to the average of the activity data present in the training set. A significant pharmacophore model might obtain when the difference between the null and the fixed cost value is large. If the value between the 40–60% and greater than 60% bits represents that the pharmacophore has 75–90% and greater than 90% probability of correlating the data, respectively. Other two parameters also play a major role to determine the quality of the hypothesis, such as configuration cost and error cost. The configuration cost also known as entropy cost based on the complexity of the pharmacophore hypothesis space and should have a value less than 17. The error cost is purely depends on RMSD between the predicted and experimental activities of training set compounds. The RMSD represents the quality of correlation between predicted and experimental values. The best pharmacophore should have highest cost difference, eminent correlation coefficient and lowest RMSD.

#### 3.2.2. Fischer’s Randomization Test

The predicted power of hypothesis is determine by evaluating the statistical significance using cross-validation procedure derived from Fischer’s randomization method. In this method the training set molecules were modified by scrambling the activity values for all the compounds. These stochastic spreadsheets yield a number of hypotheses without any statistical significance. To achieve the 98% statistical significance level, 49 random spreadsheets were generated. For 49 random spreadsheets, randomization will give 49 different hypotheses and its corresponding values like total cost, null cost, RMSD, correlation coefficient *etc*. When comparing these values with the best hypothesis, it should have a highest cost difference, lowest total cost, good correlation coefficient and lowest RMSD. This will further support the statistical significance of the best hypothesis.

#### 3.2.3. Test Set

The best pharmacophore model which was selected based on Debnath’s has chosen to estimate the activity of the test set. Test set contains wide range of activity values and structurally diverse compounds from the training set molecules and classified as highly active, moderately active and low active compounds based on its activity values. It was used to determine whether the selected best pharmacophore model has the capability to estimate the accurate activity value for the external molecules other than training set.

#### 3.2.4. Decoy Set

The enrichment factor for best pharmacophore model is calculated to prove the specificity and selectivity of model. A decoy set consists of 1300 molecules which includes 13 active molecules of 11βHSD1 inhibitors. Decoy set was used to check how well the selected best hypothesis was able to discriminate the active 11βHSD1 inhibitor compounds from other molecules, based on parameters such as total number of compounds in the hit list (H_t_), number of active percent of yields (%Y), percent ratio of actives in the hit list (%A), enrichment factor (EF) and goodness of fit (GH) was calculated using the Ligand pharmacophore mapping protocol. Equations (1) and (2) were used to calculate the EF and GH:

(1) $$\text{EF}=[({\text{H}}_{\text{a}}\times \text{D})/({\text{H}}_{\text{t}}\times \text{A})]$$

(2) $$\text{GH}=[({\text{H}}_{\text{a}}/4{\text{H}}_{\text{t}}\text{A})\;(3\text{A}+{\text{H}}_{\text{t}})\times (1-(({\text{H}}_{\text{t}}-{\text{H}}_{\text{a}})/(\text{D}-\text{A}))]$$

where “H_a_” is the total number of actives molecules in the hit list, “D” is total number of molecules in the decoy set and “A” is the total number of actives in the decoy set.

### 3.3. Virtual Screening

Virtual screening of databases can be used to validate the quality of selected pharmacophore model which was generated based on the known inhibitory activity value of compounds as well as to pick the novel and potent molecule which satisfy all critical chemical features of the hypothesis for further drug development. Here, the Hypo1 comprises of four chemical features was used as 3D query to search a chemical databases like NCI [29] (~200,000 compounds), Maybridge [30] (~60,000 compounds), and Chembridge [31] (~50,000 compounds) databases. For searching databases, Fast Flexible search algorithm was used to retrieve the new scaffolds for 11βHSD1 inhibitors. The drug-like property calculation was performed by applying Lipinski’s rule of five [32] and ADMET. Lipinski’s rule of five is a simple model to forecast the absorption and intestinal permeability of a compound. According to the rule of five, compounds are considered likely to be well absorbed when they possess LogP less than 5, molecular weight less than 500, number of hydrogen bond donors less than 5, number of hydrogen bond acceptors less than 10 and number of rotatable bonds less than 10. Mainly blood brain barrier (BBB), solubility and absorption criteria’s were focused on ADME, if the molecules have the level of 3 and 0 for solubility and absorption, respectively, these values represents that the molecules have good solubility and absorption. The drug should not cross the BBB, hence the level “3” was selected means low penetration of BBB.

### 3.4. Molecular Docking

Molecular docking method can be classified into two parts: search strategy and scoring function. The conformational selection of compounds plays a high impact for the feasibility for the binding mode. GOLD 4.1 [33] (the Cambridge Crystallographic Data Centre, Cambridge, UK, 2009) performs docking of flexible ligands into protein with partial flexibility in the neighborhood of the active site based on a genetic algorithm [34]. This method allows a partial flexibility and full flexibility for protein and ligand, respectively. Ten poses for each ligand were generated and ranked by GOLD fitness score. Three dimensional crystal structure model of 11βHSD1-piperidyl benzamide complex [PDB ID: 3FRJ] was selected from Protein Data Bank [35] (PDB, www.rcsb.org) to perform the docking experiments. The interaction sphere is center in the active site and delimited by an 8 Å radius was chosen by XYZ coordinates of the co-crystal in 11βHSD1. For each of the 100 independent GA runs, a maximum number of 100,000 GA operations were performed on a set of five groups with a population size of 100 individuals. Default cut off values of 2.5 Å for hydrogen bonds and 4.0 Å for the van der Waals distance were applied. The RMSD values for the docking calculation are based on the RMSD matrix of the ranked solutions. The hit molecules which had been screened from the virtual screening and 4 active and 1 moderately active compounds from the training set were docked and evaluated using the scoring function Gold fitness score and rank binding affinities. The novel scaffolds for 11βHSD1 were selected based on the Gold score as well the appropriate interactions with the critical residues.

### 3.5. Density Functional Theory

The structural-activity relationship has been used to study, the better understanding of the interactions between the protein and ligand, to determine the electronic properties using quantum chemistry calculation by density functional theory. Herein, we have utilized the Gaussian03W molecular package [36], invoking gradient geometry optimization [37]. Geometries of the models have been first optimized with full relaxation on the potential energy surfaces at the HF/6-31G(d,p) level and the resultant geometries have been used as inputs for further calculations at the DFT (B3LYP) level to calculate the HOMO and LUMO for most active training set compound along with some of identified database hit compounds. The selection of database hit compounds based on their binding mode, molecular interaction at protein active site was only taken. Through we have calculated the electronic properties for the 11βHSD1 inhibitors as well as leads. HOMO and LUMO are very important parameters for quantum chemistry. We can determine the way the molecule interacts with other species; hence, they are called the frontier orbital’s. HOMO, which can be thought the outermost orbital containing electrons, tends to give these electrons such as an electron donor. On the other hand; LUMO can be thought the innermost orbital containing free places to accept electrons [38]. Owing to the interaction between HOMO and LUMO orbital of a structure, transition state transition of π-π* type is observed with regard to the molecular orbital theory [39]. Therefore, while the energy of the HOMO is directly related to the ionization potential, LUMO energy is directly related to the electron affinity. Energy difference between HOMO and LUMO orbital is called as energy gap (ΔE) that is an important stability for structures [40]. In our study, we compared the different structural features of hits compounds with the most active training set compound as well as to identify the better inhibitory activity toward 11βHSD1.

## 4. Conclusions

11βHSD1 is a therapeutic target for type 2 diabetes that stimulates the interest of many pharmaceutical companies and researchers. 11βHSD1 will be of therapeutic benefit by lowering glucose output and increasing the insulin sensitivity. From the results of this study we suggest that Hypo1 has the capability of predicting the activities over a wide variety of scaffolds and showed distinct chemical features that may be responsible for activity of inhibitors. Hypo1 was generated based on 30 compounds in the training set consisting of 1-HBA, 1-Hy-Ali, 2-RA and indicates that these are the critical features of 11βHSD1 inhibitors. Fischerβs randomization method indicated that the Hypo1 did not come by chance. Test set validation confirmed that the Hypo1 is able to accurately differentiate the active from the inactive compounds, as well as a high correlation coefficient of 0.93 and decoy set methods showed a high GH (0.73) as well as EF (7.5), indicating its quality. Thus, Hypo1 was used as a 3D query in the virtual screening process to screen large databases like NCI, Maybridge, and Chembridge and sorted 165 molecules based on the maximum fit value of (11), Lipinski’s Rule of Five and ADMET. These molecules were subjected to docking studies to find the suitable orientation of compounds in the active site of 11βHSD1. Docking results revealed that 47 compounds showed a high Gold Score of greater than 60, 17 out of 47 molecules showed an hydrogen bond interaction with Tyr183, Ser170 or Ala172. These 17 hit molecules as well as the few active compounds of 11βHSD1 were used to compute the DFT to calculate the electronic properties of the hit compounds. By comparing the values of HOMO, LUMO and the energy gap between the HOMO and LUMO revealed that five hit compounds had good electronic properties when compared with the active 11βHSD1 inhibitors. Thus these five hit compounds could be useful to design the inhibitors of 11βHSD1 with greater selectivity. Hypo1 therefore will help in the identification or design of potent 11βHSD1 inhibitors for further biological evaluation and optimization.

## Figures and Tables

**Table 1:** Information of statistical significance values measured in bits for the top ten hypotheses as a result of automated 3D-QSAR pharmacophore generation.

Hypo No.	Total Cost	Cost Difference a	RMS	Correlation	Features b			Max. Fit

					HBA	Hy-Ali	RA	
Hypo1	133.91	157.30	1.21	0.94	1	1	2	11.81
Hypo2	136.12	155.09	1.26	0.93	1	1	2	11.09
Hypo3	136.85	154.36	1.26	0.93	1	1	2	12.51
Hypo4	142.56	148.65	1.49	0.91	1	1	2	10.57
Hypo5	153.2	138.01	1.69	0.88	1	1	2	11.09
Hypo6	158.37	132.84	1.85	0.85	1	2	1	8.28
Hypo7	161.76	129.45	1.86	0.85	1	1	2	11.01
Hypo8	164.01	127.20	1.95	0.84	1	1	2	8.67
Hypo9	164.08	127.13	1.79	0.87	1	2	1	13.13
Hypo10	165.89	125.32	1.98	0.83	1	2	1	8.86

a Cost difference between the null and the total cost;

b Abbreviation used for features; HBA, Hydrogen Bond Acceptor; Hy-Ali, Hydrophobic Aliphatic; RA, Ring Aromatic.

**Table 2:** Actual and estimated activity of the training set molecules based on the pharmacophore model Hypo1.

Compound No.	Fit Value a	Exp.IC_50_ nM	Pred.IC_50_ nM	Error	Exp. Scale b	Pred. Scale b
1	9.39	0.1	0.61	6.1	+++	+++
2	8.49	0.88	4.9	5.5	+++	+++
3	9.5	1.1	0.48	−2.3	+++	+++
4	9.4	1.1	0.6	−1.8	+++	+++
5	8.54	1.4	4.3	3.1	+++	+++
6	8.71	1.7	2.9	1.7	+++	+++
7	9.45	2	0.53	−3.8	+++	+++
8	8.62	2.4	3.6	1.5	+++	+++
9	8.03	2.5	14	5.6	+++	++
10	8.72	3.6	2.9	−1.3	+++	+++
11	8.52	3.8	4.5	1.2	+++	+++
12	8.17	4	10	2.5	+++	++
13	8.71	4.6	3	−1.6	+++	+++
14	8.71	4.7	3	−1.6	+++	+++
15	8.55	5.5	4.3	−1.3	+++	+++
16	8.6	6.5	3.8	−1.7	+++	+++
17	8.2	10	9.5	−1.1	++	+++
18	7.33	11	71	6.4	++	++
19	7.84	16	22	1.4	++	++
20	7.95	17	17	−1	++	++
21	7.87	35	20	−1.7	++	++
22	8.46	36	5.2	−6.9	++	+
23	7.7	79	30	−2.7	++	++
24	6.91	110	180	1.7	+	+
25	5.84	500	2200	4.4	+	+
26	5.61	1000	3700	3.7	+	+
27	5.48	13,000	5000	−2.6	+	+
28	5.49	20,000	4900	−4.1	+	+
29	5.4	20,000	5900	−3.4	+	+
30	5.4	53,000	6000	−8.8	+	+

a Fit value indicates how well the features in the pharmacophore overlap the chemical features in the molecule. Fit = weight × [max (0, 1 − SSE)] where SSE = (D/T)^2^, D = displacement of the feature from the center of the location constraints and T = the radius of the location constraint sphere for the feature (tolerance);

b Activity scale: IC_50_ < 10 nM = +++ (highly active); 10 nM ≤ IC_50_ < 100nM = ++ (moderately active); IC_50_ ≥ 100 nM = + (low active).

**Table 3:** Experimental and predicted IC_50_ data values of 20 test set molecules against Hypo1.

Compound No.	Fit Value a	Exp. IC_50_nM	Pred. IC_50_nM	Error	Exp. Scale b	Pred. Scale b
1	7.273	2.90	2.94	+1.01	+++	+++
2	8.711	7.20	8.66	+1.20	+++	+++
3	5.202	11	17.54	+1.59	++	++
4	6.608	22	41.42	+1.88	++	++
5	6.549	74	80.70	+1.09	++	++
6	6.721	123	424.94	+3.45	+	+
7	7.936	218	257.82	+1.18	+	+
8	5.82	282	287.97	+1.02	+	+
9	6.769	381	373.32	−1.02	+	+
10	6.181	381	427.44	+1.12	+	+
11	6.255	850	842	−1.01	+	+
12	5.603	980	997.78	+1.02	+	+
13	8.243	1730	1182.70	−1.46	+	+
14	6.009	2050	2079.91	+1.01	+	+
15	7.563	2130	2824.87	+1.33	+	+
16	5.729	2250	1791.88	−1.26	+	+
17	5.862	2350	1096.74	−2.14	+	+
18	5.977	4670	3576	−1.31	+	+
19	6.552	10000	1850.38	−5.40	+	+
20	4.857	10000	21026	+2.10	+	+

a Fit value indicates how well the features in the pharmacophore overlap the chemical features in the molecule. Fit = weight × [max (0, 1 − SSE)] where SSE = (D/T)^2^, D = displacement of the feature from the center of the location constraints and T = the radius of the location constraint sphere for the feature (tolerance);

b Activity scale: IC_50_ < 10 nM = +++ (highly active); 10 nM ≤ IC_50_ < 100nM = ++ (moderately active); IC_50_ ≥ 100 nM = + (low active).

**Table 4:** Statistical parameters from screening of the decoy sets of molecules.

No.	Parameter	Values
1	Total number of molecules in database	1300
2	Total number of actives in database (A)	13
3	Total number of hit molecules from the database (H_t_)	12
4	Total number of active molecules in hit list (H_a_)	9
5	% yield of actives[(H_a_/H_t_) × 100]	75
6	% Ratio of actives [(H_a_/A) × 100]	69.23
7	Enrichment Factor (EF)	7.5
8	False negatives [A − H_a_]	4
9	False Positives [H_t_ − H_a_]	3
10	Goodness of fit score (GH)	0.73

**Table 5:** The Gold fitness scores for 17 leads from GOLD docking.

Name	Structure	Gold Score	S(hb_ext)	S(vdw_ext)
Compound 23516	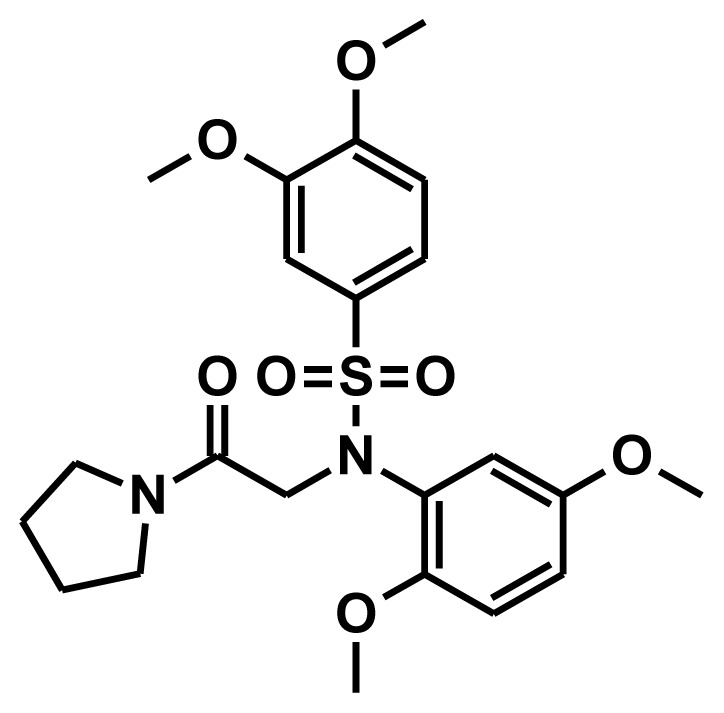	62.61	2.05	44.05
HTS 05706	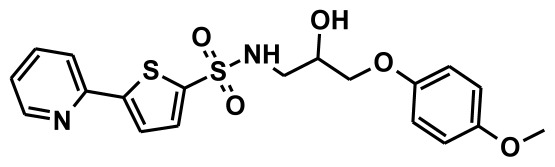	64.01	0.61	46.11
NCI0022030	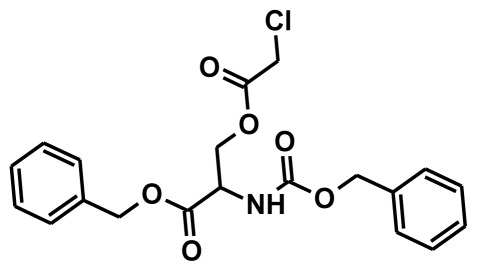	64.11	8.45	40.49
NCI0025130	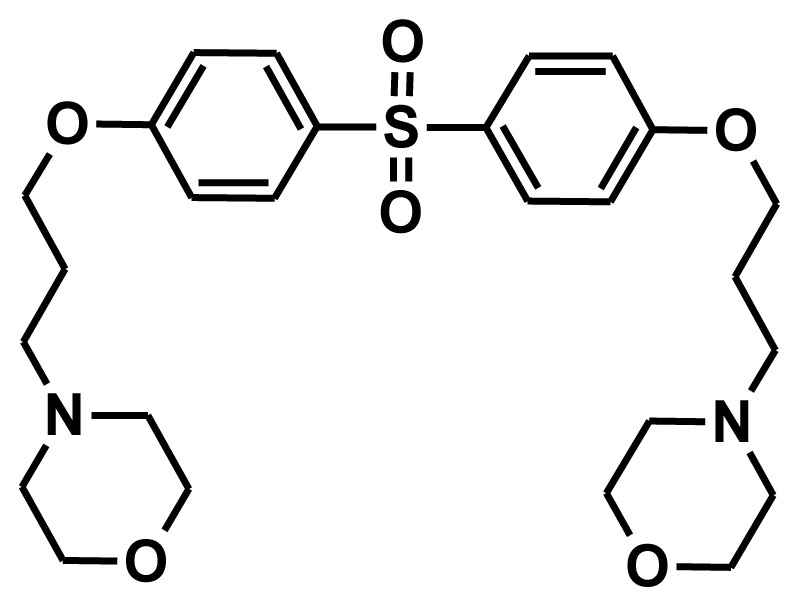	64.75	4.52	43.81
KM 10378	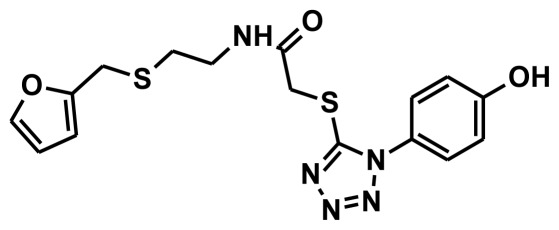	65.43	1.45	46.56
SPB 02668	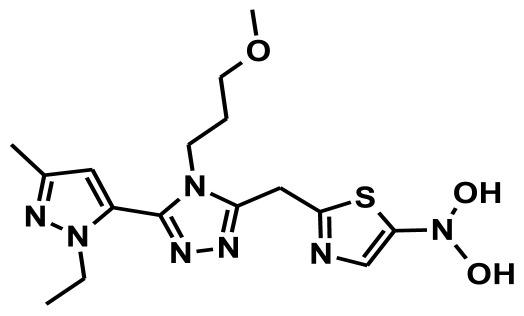	65.93	7.07	42.82
RJC 03502	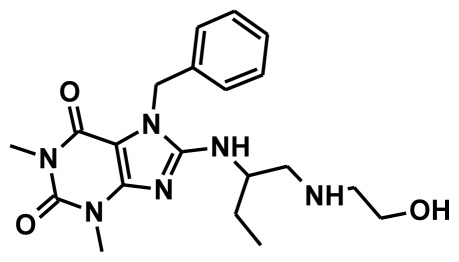	66.29	1.08	47.45
JFD 03179	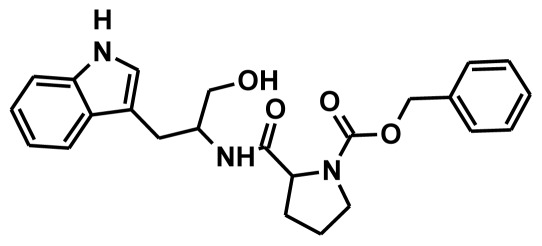	66.84	1.42	47.62
SCR 00436	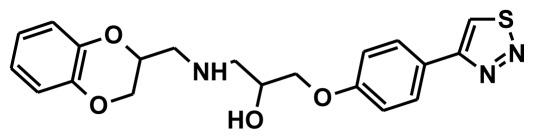	67.04	0.15	48.68
SCR 00883	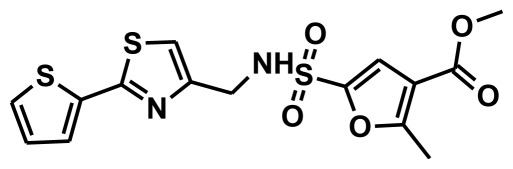	67.87	3.32	46.94
Compound 10978	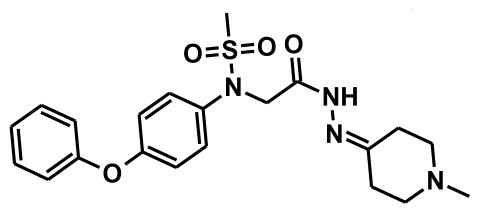	68.04	1.75	48.25
HTS 07455	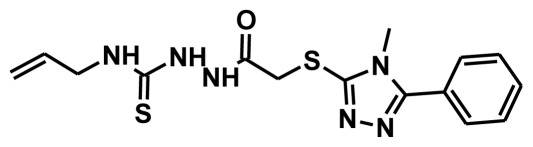	72.24	2.23	50.94
HTS 08985	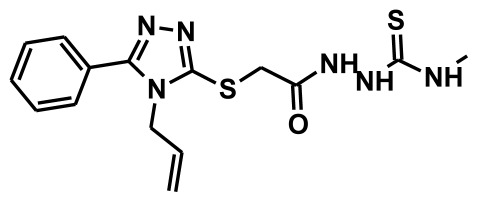	75.27	3.00	52.59
NCI0050873	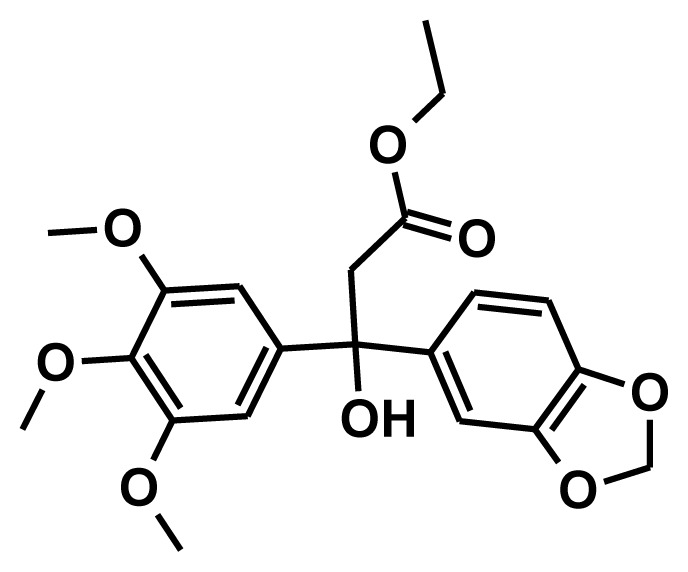	75.46	6.03	50.53
KM 06091	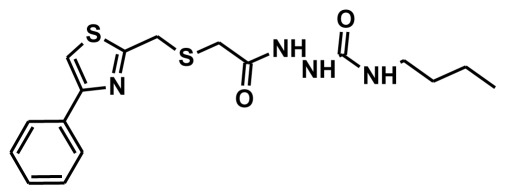	75.56	4.00	52.07
NCI0031862	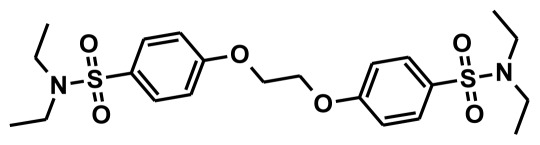	76.17	2.28	53.78
SPB 07954	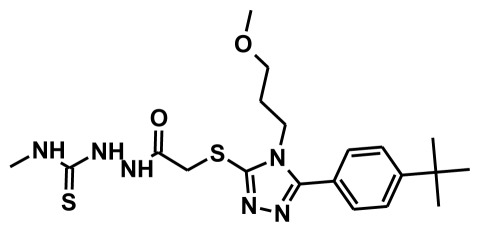	83.04	0.62	59.95

**Table 6:** Comparison of highest and lowest molecular orbital values for hit leads and active inhibitors of 11βHSD1.

Name	Structure	HOMO	LUMO	ΔE a
KM 06091	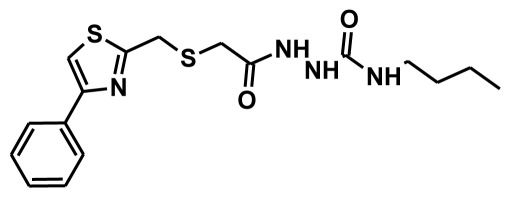	−8.31	−0.93	7.37
HTS 08985	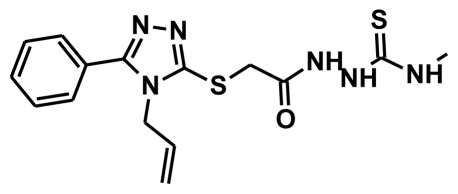	−8.44	−0.92	7.52
Compound 10978	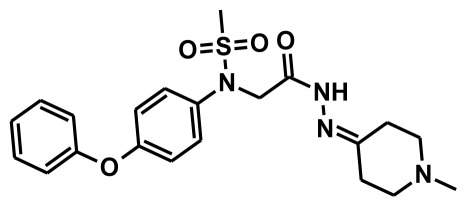	−8.47	−0.78	7.69
HTS 07455	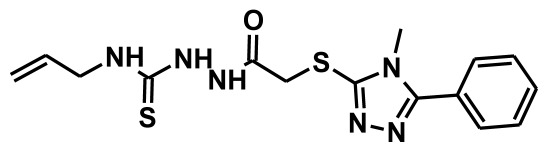	−8.48	−0.63	7.84
SPB 07954	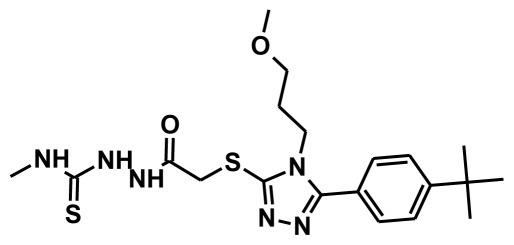	−8.51	−0.79	7.71
Training9	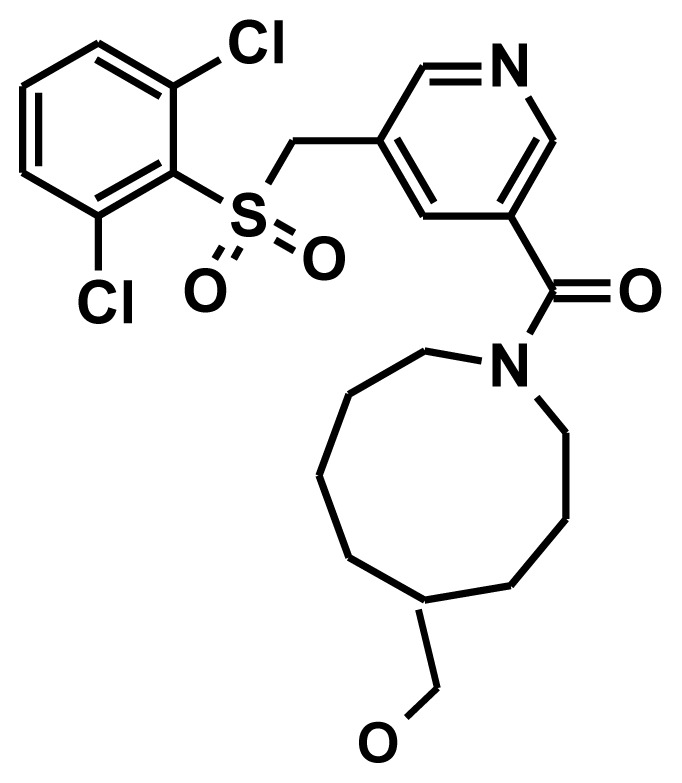	−8.56	−1.20	7.35
NCI0050873	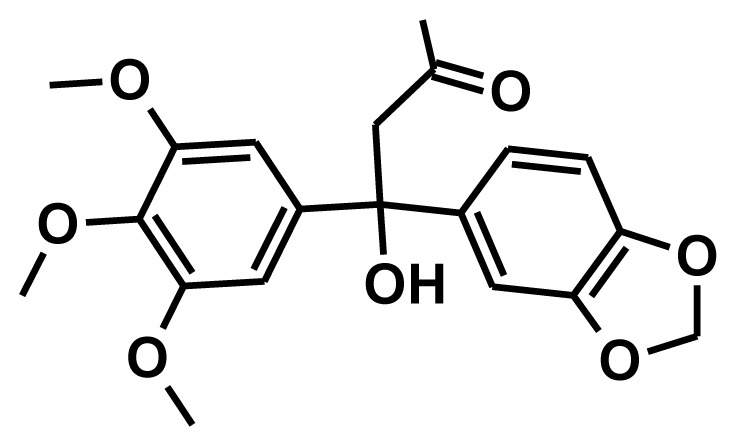	−8.73	−0.07	8.65
Training12	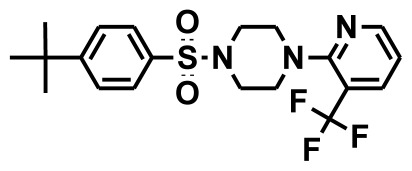	−8.74	−0.76	7.97
Training17	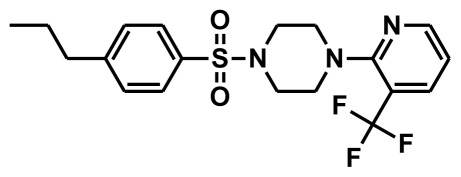	−8.89	−0.73	8.16
HTS 05706	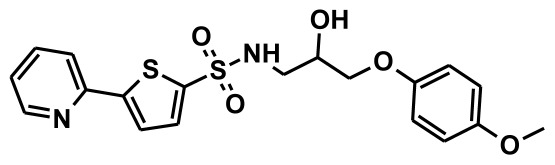	−8.93	−1.72	7.21
KM10378	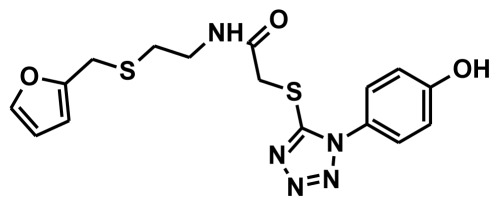	−8.95	−1.72	7.21
SCR 00883	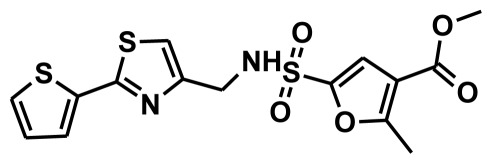	−9	−1.18	7.82
SPB02668	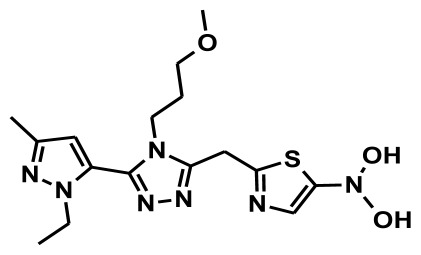	−9.04	−1.86	7.18
JFD03179	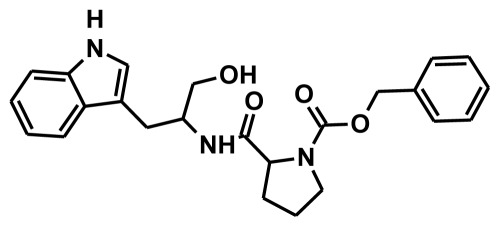	−9.07	−0.87	8.20
NCI22030	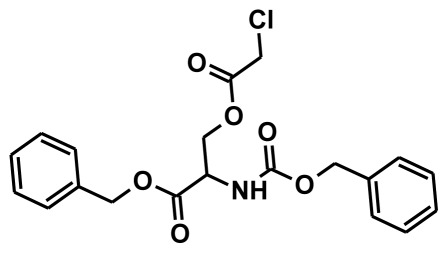	−9.11	−0.16	8.71
NCI0025130	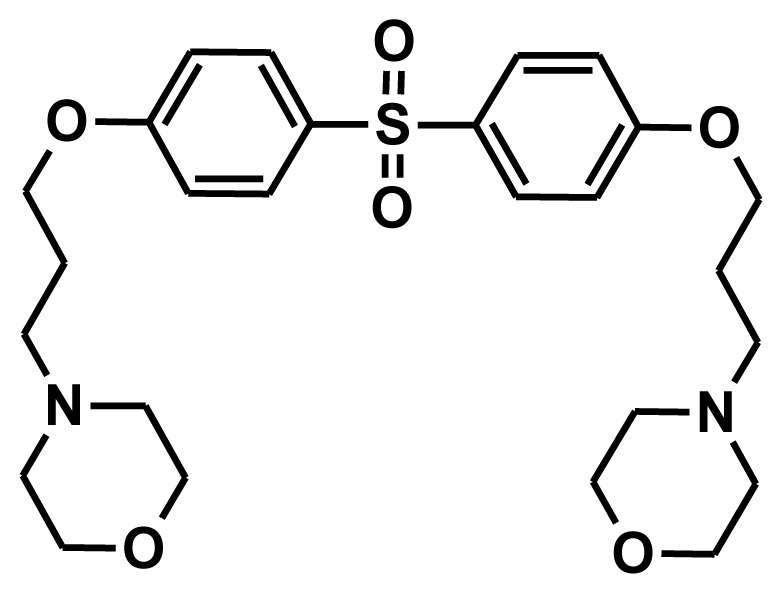	−9.32	−0.61	8.71
NCI0031862	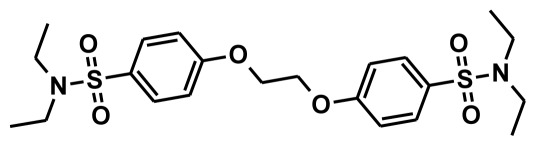	−9.84	−1.11	8.73
RJC03502	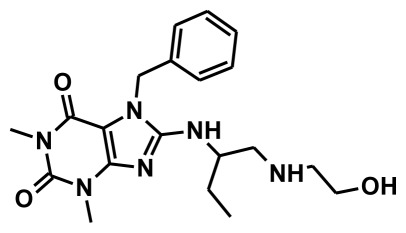	−9.96	−1.44	8.52
SCR00436	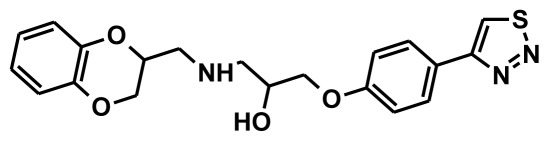	−9.56	−1.81	8.46

a Energy difference between HOMO and LUMO orbital.
